# Health-Related Quality of Life with Iatrogenic Inferior Alveolar Nerve Injuries Treated with Photobiomodulation: A Comparative Study

**DOI:** 10.3390/jcm13237237

**Published:** 2024-11-28

**Authors:** João Mendes de Abreu, Tiago Nunes, Pedro A. Almiro, José Figueiredo, Ana Corte-Real

**Affiliations:** 1 Faculty of Medicine, University of Coimbra, 3000-548 Coimbra, Portugal; jruideabreu@ulscoimbra.min-saude.pt (J.M.d.A.); tiago.nunes@fmed.uc.pt (T.N.); jpf@mail.telepac.pt (J.F.); 2 Stomatology Service, Head, Neck & Skin Surgery Department, Coimbra Hospital and University Centre, 3004-561 Coimbra, Portugal; 3 Clinical and Academic Centre of Coimbra, 3004-531 Coimbra, Portugal; 4 Forensic Dentistry Laboratory, Faculty of Medicine, University of Coimbra, 3000-548 Coimbra, Portugal; 5 Center for Research in Neuropsychology and Cognitive Behavioral Intervention, Faculty of Psychology and Education Sciences, University of Coimbra, 3000-115 Coimbra, Portugal; pedroarmelimalmiro@gmail.com; 6 Research Centre for Psychology, Autonomous University, 1169-023 Lisbon, Portugal

**Keywords:** photobiomodulation, low-level light therapy, inferior alveolar nerve injuries, iatrogenic disease, third molar, orthognathic surgery, dental implants, quality of life, health-related quality of life

## Abstract

**Background/Objectives**: Photobiomodulation therapy (PBM) creates a biostimulatory or modulatory effect, promoting tissue regeneration and improving patients’ health-related quality of life (HRQoL). PBM has shown promise as an effective treatment and management strategy for peripheral nerve injuries, including inferior alveolar nerve (IAN) damage. This study aims to assess the impact of PBM on HRQoL in patients with iatrogenic IAN injuries. **Methods**: A prospective study was implemented to investigate the research question. PBM treatments were administered weekly, with patient discharge contingent upon either complete recovery or stabilization of associated signs and symptoms. HRQoL was assessed using the EQ-5D-5L questionnaire at the initial and final appointments. **Results**: The study included 71 participants, divided into 3 groups based on etiology: inferior third molar surgery, mandibular orthognathic surgery, and dental implant surgery. The results showed a widespread reduction of symptoms along with a statistically significant improvement (*p* < 0.001) across four of the five dimensions of the EQ-5D-5L questionnaire, as well as in patients’ perceived health levels in both Groups I and II. Group III patients also demonstrated a notable improvement; however, due to the small sample size, statistical analysis was not conducted for this group. **Conclusions**: PBM demonstrated a comparable ability to enhance HRQoL and alleviate symptoms in patients with IAN injuries within different etiologies. These findings underscore the effectiveness of the protocol used in this study and highlight potential avenues for expanding research in this field.

## 1. Introduction

### 1.1. Photobiomodulation

Photobiomodulation therapy (PBM) is a clinical intervention based on the use of non-ionizing light sources, operating in the visible and infrared spectrum. It aims to achieve a biostimulatory or modulatory effect, providing therapeutic benefits such as pain relief, reduction of inflammation, and modulation of the immune system, while promoting wound healing and tissue regeneration [1,2]. PBM can exert its effects at the intracellular and extracellular levels by increasing the synthesis, production, and release of molecules such as adenosine triphosphate (ATP), serotonin, and β-endorphins. Furthermore, there is a significant reduction in C-fiber neuron activity and a decrease in bradykinin secretion. As a result, the enhancement of anti-inflammatory processes in the affected area accelerates tissue repair, promotes bone regeneration, and supports the restoration of neural function [1,3,4,5,6]. Based on these premises, the application of PBM therapy has proven to be an effective treatment modality for the management of a variety of pathological conditions and iatrogenic injury [7], including musculoskeletal disorders [8], chemotherapy side effects [9], burning mouth syndrome [10], and peripheral nerve injuries such as inferior alveolar nerve (IAN) damage [3,4,6,11,12].

### 1.2. Iatrogenic Inferior Alveolar Nerve Injury

The trigeminal nerve (cranial nerve V) is the primary sensory nerve of the head, and it branches into three major divisions: the ophthalmic (V1), maxillary (V2), and mandibular (V3) nerves [13]. Trigeminal nerve injuries can arise from various etiologies, with iatrogenic injuries being the most common. Furthermore, it is established that the third division of the trigeminal nerve is more susceptible to damage, particularly the IAN [6,14]. This occurrence is primarily attributable to anatomic considerations [13], which can present a significant challenge when undertaking common widespread procedures such as mandibular regional anesthesia (0.004%), wisdom tooth extraction (0.5% to 8%), implant placement (0–24%), endodontic treatment (0.086%), or even orthognathic surgery, such as a bilateral sagittal split osteotomy (BSSO) of the mandible (70%) [6,14,15,16,17,18,19,20].

Despite up to 90% of all IAN injuries being transient and resolving within eight weeks, a small proportion of cases may result in corporal damage [6,12,15,16,20]. Patients with these injuries may experience paresthesia, dysesthesia, or even neuropathic pain, contributing to a diminished health-related quality of life (HRQoL), as well as future litigation. Some patients can also present functional limitations such as drinking and chewing and improper oral hygiene. They may also report self-harming injuries, such as unintentionally biting their lower lip due to sensory loss [6,12,14,21,22].

Regarding the management of these injuries, although some authors have proposed complex surgical procedures such as external neurolysis, the supporting evidence remains uncertain. However, immediate neurorrhaphy, especially when repairing transected IAN during sagittal split osteotomies, and autologous nerve grafting may be notable exceptions to this ambiguity [3,12]. In terms of a pharmacological approach, administering corticosteroids during the perioperative period and the patient’s recovery has proven beneficial. By reducing edema and neural inflammation, they enhance nerve healing and improve overall recovery outcomes [3,4]. Neurotropic B vitamins are likewise suggested as a potential therapeutic option, given their role in establishing optimal conditions for nerve regeneration. However, further studies are required to fully understand the extent and influence of this therapeutic approach [3,23]. Biological alternatives such as growth factors and mesenchymal stem cells have also been proposed as complements to existing treatments for nerve tissue recovery and regeneration. However, the evidence supporting this approach remains limited [3,4,24].

More recently, PBM has emerged as a promising approach for the treatment and management of peripheral nerve injury, including IAN affectation, due to its proven efficacy, lack of side effects, and beneficial biological effects. This procedure improves nerve regeneration while reducing symptoms, promoting positive changes in the affected dermatome, and enhancing HRQoL [3,4,6,11,12]. However, a standard PBM protocol for the treatment of IAN injuries is still absent given the diversity of methodologies, significant variations in parameters (timeline, number of sessions, irradiation variables) between experimental settings, and potential biases. Moreover, recent reviews and meta-analyses consistently highlight the need for further clinical trials and more robust evidence to validate their efficacy [6,25,26]. Other shortcomings in the literature are the lack of comparison between different etiologies of IAN injury, as well as the evaluation of PBM impact on HRQoL, as existing studies focus on thermal discrimination, two-point discrimination, and pinprick testing while using visual analog scales (VAS) to assess outcomes [6,25,26].

### 1.3. Objectives

This study aims to evaluate the benefits of PBM on HRQoL in patients with iatrogenic IAN injuries of different etiologies through a comparative study. Unlike previous research, this study avoids predetermined session limits and uses a practical outpatient protocol, emphasizing a personalized approach that balances patient convenience with optimized clinical outcomes for effective and minimally burdensome treatment while overcoming previous limitations. Therefore, we consider the null hypothesis that the implemented PBM protocol does not provide any benefit to patients with an iatrogenic injury to the IAN.

## 2. Materials and Methods

### 2.1. Study Design and Institutional Approval

An interventional comparative study was designed to address the following research questions: Can PBM improve the HRQoL of patients with iatrogenic IAN injury? Do patients benefit equally from a viable outpatient/private clinic protocol compared to more intensive experimental regimens?

Institutional approval was granted by the Ethics Committee of the Faculty of Medicine of the University of Coimbra, Portugal (reference number CE-028/2022). An informed consent form was provided to and signed by all patients in this study. All procedures adhered to the ethical tenets of the World Medical Association’s Declaration of Helsinki [27].

### 2.2. Patient Selection

The study population was recruited from the Dentistry, Stomatology, and Maxillofacial Outpatient Departments at the Clinical and Academic Centre of Coimbra between 1 July 2022 and 30 June 2024. Based on the Cochrane Intervention Review findings by Coulthard et al. [12], the inclusion criteria comprised adult patients with persistent (>8 weeks) iatrogenic IAN injury. Patients were excluded from the study according to the following criteria: absence of iatrogenic IAN injury at the initial evaluation; bilateral IAN injury; initial procedure driven by infectious, cystic, or oncologic lesions; occurrence of repaired or unrepaired, partial or complete, neurotmesis during the procedure; the presence of other surgical complications (e.g., infection) or the need for reintervention; pregnancy; previously diagnosed conditions that could affect HRQoL assessment (e.g., depression, fibromyalgia); overlapping signs and symptoms associated with IAN damage that were present before surgery (e.g., paresthesia, hypoesthesia/anesthesia, difficulty in chewing or unintentional biting, and challenges with oral hygiene). Patients were also excluded from the study if unable to understand the text of the informed consent form and questionnaire or failed to adhere to the previously established treatment schedule.

### 2.3. Patient Assessment

**Figure 1:**
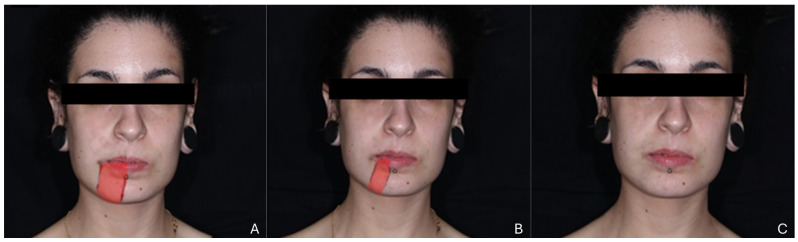
Photographic record of the evolution of a patient’s sensory deficit through the mapping of the affected regions following pinprick testing. (**A**) First appointment. (**B**) During PBM. (**C**) Last appointment.

All patients who met the inclusion criteria were referred to a PBM appointment. A clinical history was performed, and the exclusion criteria were applied. Subgroups were created according to the underlying injury etiology. The presence of any specific symptoms of IAN damage (e.g., paresthesia, hypoesthesia, anesthesia, dysesthesia) was recorded and monitored in the subsequent appointments. Furthermore, a physical assessment to evaluate the sensory deficit was conducted, comprising a pinprick test and mapping of the affected region. During the procedure, the patient’s eyes were kept closed, and a sharp dental probe was placed in direct contact with the labial and mental skin, with the operator applying light pressure. Subsequently, the patient was requested to identify and touch the probed area. The surface was considered affected if the pinprick point was misidentified or not identified. Following the mapping, the area was calculated and expressed in cm^2^, with the value obtained rounded up to the nearest whole number (Figure 1). The contralateral non-affected side was used as a control. The process was repeated at the beginning of each consultation. HRQoL was assessed using the Portuguese version of the EQ-5D-5L [28] at the first and last visits. Satisfaction with treatment and likelihood to recommend treatment were likewise evaluated using an 11-point Likert scale after the patient’s discharge.

### 2.4. Intervention

**Figure 2:**
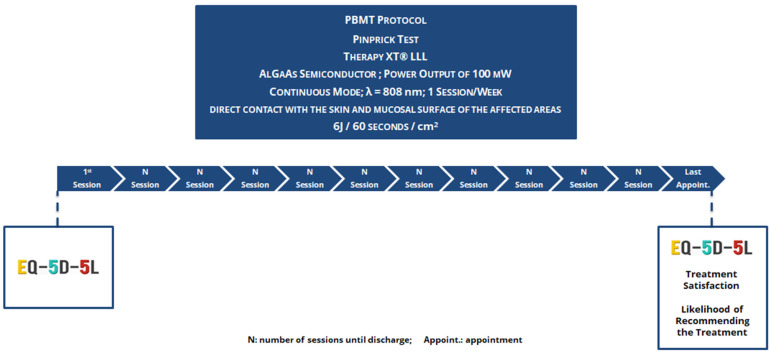
PBM experimental protocol, combining comprehensive patient assessment and predefined intervention parameters.

PBM was performed by irradiating the extraoral affected regions and their intraoral counterparts at weekly intervals without a previously imposed limit of sessions. The patient’s discharge was contingent upon the complete resolution or stabilization of signs and symptoms, considering the absence of improvement during two separate visits. The process consisted of using a Low-Level Laser Therapy (LLLT) device, specifically Therapy XT^®^ (DMC, São Carlos, SP, Brazil), with predetermined parameters (continuous mode, λ = 808 nm, AlGaAs semiconductor, a power output of 100 mW, and dosage of 6 J/60 s/cm^2^) (Figure 2). The probe remained in contact with the tissue throughout the treatment as it moved over the pre-determined affected area. The same clinician performed all treatments, and all safety protocols were followed, including the use of appropriate personal protective equipment (PPE), during PBM sessions.

### 2.5. Outcome Assessment and Variables

The EQ-5D-5L HRQoL tool is a five-dimensional, five-point (1–5) assessment instrument that includes a visual analog scale (VAS) ranging from 0–100. It evaluates the patient’s ability to perform movement, self-care, and usual activities, as well as their perception of pain/discomfort and anxiety/depression, accompanied by their perception of their overall health status, which is quantified on a scale of 0 to 100, with higher numbers indicating better health [28]. Satisfaction and likelihood to recommend treatment were also evaluated using an 11-point Likert scale, with higher scores indicating a more favorable outcome. Additional variables considered included sex, age, sensory deficit (affected area), symptom characterization, functional complaints, time since the initial onset of symptoms, and use of IAN injury-specific prescriptions.

### 2.6. Data Analysis

The identities of the patients were protected by alphanumeric anonymization to ensure confidentiality. In that regard, the results were also independently assessed, without the involvement of the agent who carried out the clinical intervention.

A statistical study was performed with IBM SPSS Statistics^®^ software (Version 28.0.1.0 (142)). Descriptive statistics were used for the assessment of frequency, central tendency, and measures of variation. Normality was assessed using the Shapiro–Wilk test. Inferential statistics were performed using nonparametric tests, namely the Wilcoxon test (for paired samples) and Spearman’s Rho to analyze the correlation between study variables. A *p*-value of less than 0.05 was considered statistically significant.

## 3. Results

**Figure 3:**
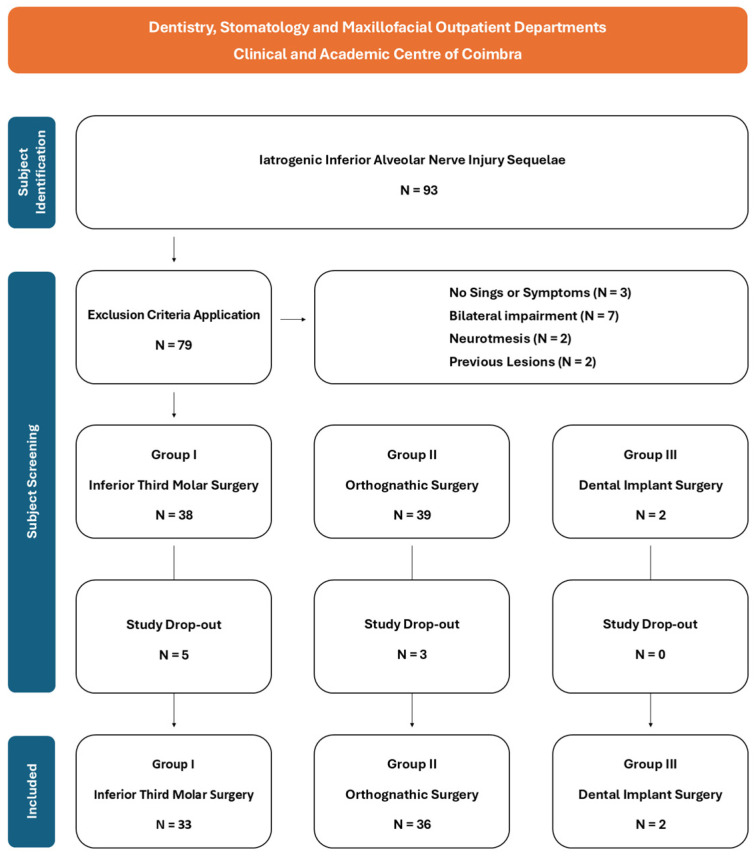
Patient selection flowchart.

From 1 July 2022 to 30 June 2024, 93 patients with persistent (>8 weeks) iatrogenic IAN injuries were referred for PBM appointments. Applying the exclusion criteria, 14 patients were disqualified from this study. The remaining 79 patients were divided into 3 cohorts according to etiology: 38 patients in Group I—Inferior Third Molar Surgery; 39 patients in Group II—Mandibular Orthognathic Surgery (all BSSO); and 2 patients in Group III—Dental Implant Surgery. A total of 71 subjects completed the study, following the exclusion of 8 dropouts (5 patients in Group I—Inferior Third Molar Surgery; 3 patients in Group II—Mandibular Orthognathic Surgery) (Figure 3). Sample characteristics according to the attributed group are displayed in Table 1.

### 3.1. Group I—Inferior Third Molar Surgery

The inferior third molar surgery group (Group I) consisted of 15 females (45.45%) and 18 males (54.55%) with a mean age of 35.7 years (±9.989), ranging from 19 to 66 years. A similar proportion was found for the extracted tooth, with 18 patients (54.55%) undergoing the extraction of the left mandibular wisdom tooth and 15 (45.45%) undergoing the extraction of the right mandibular wisdom tooth. Regarding the surgical technique, 28 patients (84.85%) underwent a more complex and invasive approach requiring osteotomy and odontosection of the tooth, 4 (12.12%) underwent osteotomy, while a single patient (3.03%) presented with IAN injury after a simple extraction.

Hypoesthesia was the primary symptom reported in 28 patients (84.85%), while 17 patients (51.52%) also reported concurrent paresthesia. Patients similarly noted significant functional complaints, with 17 (51.52%) expressing relevant unawareness of the presence of food and fluid in the affected areas, 11 (33.33%) reporting unintentional self-inflicted bite wounds, and 7 (21.21%) reporting difficulty shaving. Regarding prior targeted therapies, 16 patients (48.48%) received both corticosteroids and vitamin B, while 1 patient (3.03%) received just corticosteroids, and 5 patients (15.15%) received only vitamin B.

The mean time from the onset of the iatrogenic lesion to the first PBM session was 194.45 (±62.508) days, ranging from 108 to 412 days. On average, patients were submitted to 11.97 (±4.305) PBM sessions, ranging from 6 to 22 in total. Subjective and objective components were also evaluated, with patients reporting perceived improvement after 1.82 (±0.635) sessions compared to 1.61(±0.496) sessions required to achieve objective improvement on the pinprick test. The mean initial cutaneous affected area was 6.88 (±2.395) cm^2^, ranging from 2 to 12 cm^2^, which decreased to 0.76 (±0.936) cm^2^, ranging from 0 to 3 cm^2^, at the final evaluation.

### 3.2. Group II—Orthognathic Surgery

The orthognathic surgery group (Group II) consisted of 24 females (66.67%) and 12 males (33.33%) with a mean age of 35.19 years (±9.386), ranging from 23 to 59 years. Patients complained of right IAN involvement in 20 cases (55.56%) and left IAN involvement in the remaining 16 cases (44.44%). The surgical approach also varied, with 22 patients (61.11%) undergoing advancement BSSO, 2 patients (5.56%) undergoing retraction BSSO, and 12 patients (33.33%) receiving a combined approach.

Hypoesthesia was the most common complaint, present in 20 patients (55.56%), while 6 (16.67%) and 5 patients (13.89%), respectively, also reported concurrent and isolated paresthesia. Unawareness of the presence of food and fluid in the affected areas was the most reported functional limitation in 27 patients (75%), followed by unintentional self-inflicted biting in 9 patients (25%), 8 (22.22%) of whom were concurrent. In terms of prior directed therapies, 32 patients (88.89%) were prescribed corticosteroids, with vitamin B added in 9 cases (25%).

The mean duration from the onset of the iatrogenic lesion to the first PBM session was 427.89 (±267.76) days, with a range of 123 to 1065 days. On average, patients underwent 11.67 (±4.34) PBM sessions, with the total number of sessions ranging from 5 to 21. Both subjective and objective improvements were assessed. Patients reported perceiving improvement after an average of 1.89 (±1.01) sessions, while an average of 2.00 (±0.93) sessions were required to achieve measurable improvement on the pinprick test. The mean initial cutaneous affected area was 9.00 (±3.74) cm^2^, ranging from 2 to 16 cm^2^, which decreased to 1.39 (±3.60) cm^2^, ranging from 0 to 16 cm^2^, at the final evaluation.

### 3.3. Group III—Dental Implant Surgery

The dental implant surgery group (Group III) comprised two women, aged 36 and 41 years. The first patient received a dental implant as part of the rehabilitation process for the replacement of the mandibular right first molar (tooth 4.6), while the second patient underwent a similar procedure to replace the mandibular left second premolar (tooth 3.5).

Hypoesthesia was present in both subjects, with one also reporting concurrent paresthesia. In terms of functional limitations, both stated unawareness of the presence of food and fluid in the affected areas. As with previous therapies, 1 patient (50%) was prescribed specific medications, in this case vitamin B.

Patients received the first PBM session 65 and 72 days after the implant placement, followed by 7 and 9 sessions, respectively. Subjective improvement was noted after 1 and 2 sessions, while objective improvement was not achieved until the second session for both patients. The initial affected area was 2 and 3 cm^2^, decreasing to 0 at the final evaluation.

### 3.4. Sensory Deficit and Health-Related Quality of Life Assessment

The pinprick test demonstrated substantial clinical improvement across all three groups, with an average reduction in affected areas of 6.12 cm^2^, 7.61 cm^2^, and 2.5 cm^2^, respectively. This improvement was statistically significant in the first two groups (*p* < 0.001), as determined by the Wilcoxon test.

**Figure 4:**
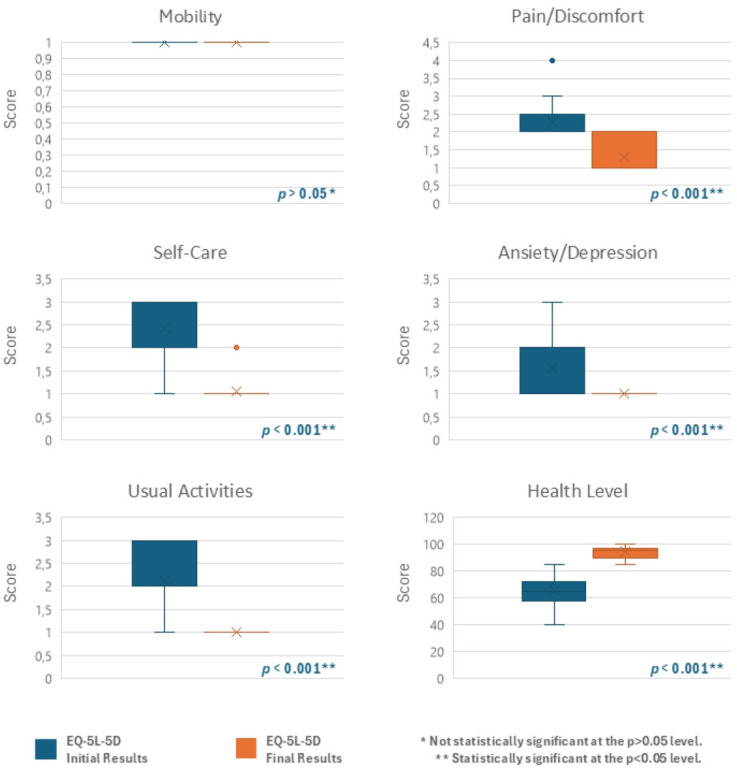
EQ-5D-5L questionnaire initial and final results—Group I (Vertical Boxplot Chart).

**Figure 5:**
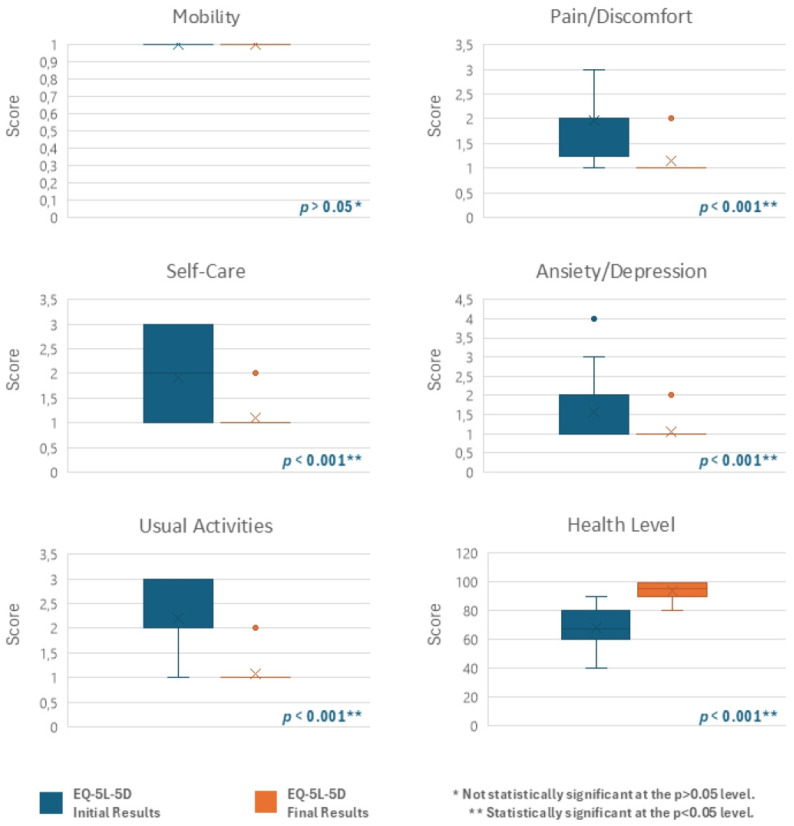
EQ-5D-5L questionnaire initial and final results—Group II (Vertical Boxplot Chart).

Similarly, patients’ HRQoL showed a meaningful improvement in all groups between the two assessment points in four dimensions of the EQ-5D-5L questionnaire: self-care, usual activities, pain/discomfort, and anxiety/depression. However, the fifth dimension, related to mobility, remained unchanged (Figure 4 and Figure 5). Patients’ perceived health, as measured by the EQ-VAS, also showed a notable improvement, with the mean score increasing by 28.94 (Group I), 25.33 (Group II), and 37 (Group III) points from the beginning to the end of the study (Figure 4 and Figure 5). These results demonstrate statistically significant differences (*p* < 0.001) as determined by the Wilcoxon Test, in groups I and II, accompanied by a relevant effect size (Table 2 and Table 3).

Furthermore, Spearman’s Rho (ρ) analysis revealed and denied statistically significant correlations between the VAS Score of the EQ-5D-5L questionnaire and other analyzed variables (Table 4 and Table 5).

Statistical analysis was not conducted for Group III due to the limited sample size.

### 3.5. Treatment Satisfaction and the Likelihood of Recommendation

Treatment satisfaction and the likelihood of recommending the treatment indicated positive outcomes across all groups. In Group I, the mean satisfaction score was 9.33 (±0.736), ranging from 8 to 10, with a recommendation score of 10. In Group II, the mean satisfaction score was 9.33 (±1.242), ranging from 5 to 10, and the mean recommendation score was 9.89 (±0.465), ranging from 8 to 10. In Group III, the scores were 9.5 and 10, respectively, on an 11-point Likert scale.

## 4. Discussion

The findings confirmed that PBM led to a statistically highly significant improvement in HRQoL, as measured by the EQ-5D-5L, in patients with iatrogenic IAN injuries in both the inferior third molar surgery and orthognathic surgery groups. Additionally, the pinprick test also revealed substantial clinical recovery in Groups I and II, with statistically highly significant differences. Patients in the dental implant surgery group also showed improvements in both HRQoL and pinprick test scores. These outcomes respond to the primary research questions, making a valuable contribution to the topic by providing an innovative and effective treatment option for patients with this pathology, regardless of their etiology.

Iatrogenic injury is the most frequently reported cause of IAN damage, with third molar extraction and orthognathic surgery usually identified as the leading causes. Additional causes include dental implant placement, mandibular regional and local anesthesia, and endodontic treatment, though the percentages vary greatly across studies in the existing literature [6,14,15,16,18,19,20,21]. Regarding dentoalveolar procedures, Kaleem et al. [14] reported that third molar extractions are associated with the highest incidence of injury (40.8%), followed by endodontic procedures (35.3%), dentoalveolar surgeries excluding third molars (20.7%), and dental implants (3.2%). Whereas Renton [29] reported that third molar extractions are the leading cause of IAN injury (60%), followed by local anesthetic injections (19%), implants (18%), and endodontic procedures (18%). In the present study, inferior third molar and mandibular orthognathic surgery accounted for over 97% of the patients (n = 69). Moreover, it also corroborates existing literature linking IAN injuries to more complex and invasive third-molar surgeries, specifically those involving osteotomy and odontosection [15]. This is evidenced by the fact that 84.85% of patients in Group I had undergone such procedures. As for the significantly lower number of patients with persistent IAN lesions associated with dental implant surgery, this discrepancy may be attributed to the rigorous preoperative evaluation and management of patients by the Dentistry and Stomatology Departments, with three-dimensional implant placement planning, assessment of bone density and cortical thickness, and adherence to guided and semi-guided surgical protocols being used in the vast majority of cases [30,31,32]. The absence of patients with persistent IAN lesions from mandibular regional anesthesia or endodontic procedures can be explained by the implementation of correct techniques accompanied by the significantly lower risk, as well as the typically transient nature of these injuries, with up to 90% of cases reportedly resolving on their own within eight to ten weeks [6,12,14,15,16,18,20].

Regarding patient age, most studies have reported that IAN injuries are most common among individuals in their third and fourth decades of life [6,25,26]. This trend was similarly observed in our study, with an average age of 35 years in Groups I and II (Group I: 35.7 years ± 9.99; Group II: 35.19 years ± 9.39). In Group III, the average age was slightly higher at 38.5 years, although it remained within literature reports. Patients with IAN injuries also experience significant dysfunctions and limitations in performing daily activities such as drinking and chewing and may even report self-inflicted biting of the lower lip and oral mucosa due to sensory loss or alteration, with a clear impact on their QoL [6,14,15,16,18,19,20,21,22]. This pattern was likewise observed among patients in this study.

In terms of treatment and management, IAN injury has historically presented significant challenges, leading to the evaluation of several classes of drugs, nutritional supplements, and therapies. However, none of the previous options is significantly superior to the others, with their efficacy and posology remaining up for debate [3,4,12,20,23,24]. This tendency was also evident in our sample, with 55 participants (77.46%) having previously been prescribed corticosteroids and/or vitamin B without fully satisfactory results. Group analysis, though, revealed marked differences, with the orthognathic surgery group presenting a notably higher corticosteroid prescription rate (32 patients, 88.89% vs. 17 patients, 51.51%). This discrepancy is likely due to the varying complexity and procedural demands of said surgical procedure, with corticosteroids commonly prescribed in these cases to reduce edema, general postoperative inflammation, and pain, thereby enhancing patient comfort [33].

More recently, PBM has shown promising success in reducing symptoms and improving QoL in patients with iatrogenic IAN lesions. However, questions remain about the relationship between its objective and subjective effectiveness and factors such as the time elapsed since injury and specific treatment protocols [6,25,26]. In the present study, patients underwent weekly sessions of PBM, with the cutaneous and corresponding intraoral affected surfaces irradiated in continuous mode, with an infrared wavelength (λ = 808 nm), a power output of 100 mW, and a dosage of 6 J/60 s/cm^2^. Discharge was based on the complete resolution or stabilization of signs and symptoms, along with the absence of further improvement over two consecutive appointments. This approach allowed for more reliable results, as opposed to studies where a fixed number of sessions was predetermined [6,25,26], thus limiting a potential late recovery. Subjective improvements were first observed after an average of 1.82 sessions (±0.64) in Group I, 1.89 sessions (±1.01) in Group II, and 1.5 sessions in Group III. Objective improvements similarly tended to appear within two sessions, with averages of 1.61 sessions (±0.50) in Group I and 2 sessions in both Group II (±0.93) and Group III. The mean time from the onset of the iatrogenic lesion to the first PBM session was 194.45 (±62.51) days in Group I, 427.89 (±267.76) days in Group II, and 68.5 days in Group III. These differences can be credited to varying follow-up regimens, with significantly longer intervals between appointments in the orthognathic surgery group, specifically after the first post-operative follow-up consultation, resulting in later referrals. Regarding the number of sessions, similar results were observed in both Groups I and II, with patients receiving an average of 11.97 (±4.305) and 11.67 (±4.34) PBM sessions, respectively. Patients in Group III received 7 and 9 sessions, also with positive results, although the small sample size prevents us from extending the conclusions. Another variable analyzed across all groups was the area of skin affected, as determined by the pinprick test. Patients who underwent orthognathic surgery exhibited a considerably larger affected area. Nonetheless, a substantial reduction was observed post-PBM in all groups, with statistically significant results in Groups I and II (*p* < 0.001), resulting in final average affected areas ranging from 0 to 1.39 cm^2^. All the previous results thus reinforce the similar efficacy of PBM regardless of the etiologic factor but are also coincident with most studies dedicated to a single-etiology group of patients [6,25,26,34]. Furthermore, the use of the pinprick test to evaluate patients’ sensory deficits and their progression proved highly effective, as the data gathered aligned with the clinical improvements observed in the patients.

Patients’ HRQoL improved in four dimensions of the EQ-5D-5L questionnaire (self-care, usual activities, pain/discomfort, and anxiety/depression) in Groups I and II. This improvement was statistically significant (*p* < 0.001) and comparable between groups, with both presenting a similarly large effect size. A statistically significant (*p* < 0.001) pattern was noted as well in the EQ-VAS, which measures patients’ perception of their health, revealing mean score increases of 28.94 and 25.33, respectively. A notable improvement was also observed in Group III; however, as noted previously, no inferential statistical analysis was conducted due to the small sample size. These findings suggest that although the symptoms of IAN lesions are confined to the oral cavity and lower face, their impact on usual activities and self-care capacity can’t be ignored, with activities such as eating, maintaining oral hygiene, and also fostering social relationships and communicating severely affected [6,12,14,21,22]. Moreover, these symptoms can impact mental health and overall health perception, a conclusion also supported by Nunes et al. [34]. This suggests that while PBM is effective over time, a comprehensive, multidisciplinary approach incorporating personalized rehabilitation programs and psychological support could further enhance outcomes and provide more holistic care for these patients. As expected, no changes were observed in the fifth dimension concerning patients’ mobility, consistent with the lack of correlation between IAN injury and mobility levels. The results also underscore the effectiveness of using the EQ-5D-5L questionnaire for patients with IAN injuries, as it successfully reflects the variations in their clinical presentation. Another significant benefit of this questionnaire is its simplicity contrasting with other instruments used to assess QoL or Oral HRWoL, such as the Medical Outcomes Study Short Form Survey-36 (SF-36) [35] or the Oral Health Impact Profile (OHIP) [36], which tend to be more extensive and complicated. This statement is also shared with previous studies [10].

Additionally, the Spearman’s Rho (ρ) test identified and dismissed several statistical correlations between variables, with no specific pattern evident among the groups. Notable exceptions included the correlation between patient age and “EQ-5D-5L Perceived Health Level” and “Treatment Satisfaction”, indicating that younger patients generally achieve higher scores in both areas by the end of treatment. Likewise, a correlation was also found between “Sensory Deficit” and “Treatment Satisfaction”, demonstrating that a lower final sensory deficit is associated with higher patient satisfaction, as one should expect.

Lastly, patients’ satisfaction and likelihood of recommending the treatment similarly reflected the positive clinical outcomes and enhanced HRQoL measured by the EQ-5D-5L tool, with average scores of at least 9.33 and 9.75, respectively, across all groups. This aligns with findings from previous studies that have established a similar relationship [10].

### Limitations

Despite the success of this study, certain limitations must be considered when interpreting the results. The main constraint is the asymmetry in sample sizes between Groups I and II compared to Group III, as well as the limited number of etiologies addressed. Both factors are inherent to the design of a unicentric clinical study and could be improved through a future multicentric collaborative research effort.

Another shortcoming is the non-blinded nature of the study, which inherently increases the risk of bias. Additionally, the data collected from self-report questionnaires is subjective and may also be prone to bias. However, the incorporation of objective measures, such as the pinprick test to evaluate sensory deficits, acts as a safeguard, thereby enhancing the validity of the findings.

## 5. Conclusions

This study offers an innovative approach to treating IAN injuries of various etiologies using a standardized PBM protocol. Findings demonstrate that PBM can similarly enhance HRQoL for patients with IAN injuries while also reducing or even normalizing the associated signs and symptoms.

The results further underscore the effectiveness of a non-intensive outpatient PBM protocol, as evidenced by the low patient dropout rate and results. This outcome suggests that a weekly session schedule offers a balanced approach, minimizing disruptions to patients’ lives without compromising treatment results. Thus, proving that the utilized therapeutic approach is well-suited for routine implementation in private office or clinic settings, consequently reducing the need for patient referrals to hospital facilities.

Given the innovative methodology and promising outcomes, further research with larger sample sizes, control groups, and varied PBM parameters is essential for advancing understanding and optimizing treatment protocols. Additional studies could also deepen insights into the diverse signs and symptoms presented by patients, helping to clarify some of the observed variations.

## Figures and Tables

**Table 1:** Sample characteristics.

Variables	Group I	Group II	Group III
Sample	33	36	2
Sex	Female: 15 (45.45%) Male: 18 (54.55%)	Female: 24 (66.67%) Male: 12 (33.33%)	Female: 2 (100%)
Age (years)	$\bar{x}$ = 35.7 (±9.989) [19:66]	$\bar{x}$ = 35.19 (±9.386) [23:59]	$\bar{x}$ = 38.5 (±2.5) [36:41]
IAN Involvement	Right: 15 (45.45%) Left: 18 (54.55%)	Right: 20 (55.56%) Left: 16 (44.44%)	Right: 1 (50%) Left: 1 (50%)
Time Since Injury (days)	$\bar{x}$ = 194.45 (±62.508) [108:412]	$\bar{x}$ = 427.89 (±267.76) [123:1065]	$\bar{x}$ = 68.5 (±3.5) [65:72]
Total Number of Sessions	$\bar{x}$ = 11.97 (±4.305) [6:22]	$\bar{x}$ = 11.67 (±4.34) [5:21]	$\bar{x}$ = 8 (±1) [7:9]
Objective Improvement (Sessions)	$\bar{x}$ = 1.61(±0.496) [1:2]	$\bar{x}$ = 2.00 (±0.93) [0:4]	$\bar{x}$ = 2 (±0) [2]
Subjective Improvement (Sessions)	$\bar{x}$ = 1.82 (±0.635) [1:3]	$\bar{x}$ = 1.89 (±1.01) [0:4]	$\bar{x}$ = 1.5 (±0.5) [1:2]
Initial Sensory Deficit (cm^2^)	$\bar{x}$ = 6.88 (±2.395) [2:12]	$\bar{x}$ = 9.00 (±3.74) [2:16]	$\bar{x}$ = 2.5 (±0.5) [2:3]
Final Sensory Deficit (cm^2^)	$\bar{x}$ = 0.76 (±0.936) [0:3]	$\bar{x}$ = 1.39 (±3.60) [0:16]	$\bar{x}$ = 0 (±0) [0]

IAN: Inferior Alveolar Nerve; $\bar{x}$: Average; ±: Standard Deviation.

**Table 2:** Variation analysis of the EQ-5D-5L questionnaire results—Group I.

Group I—EQ-5D-5L	Wilcoxon Test	*p*	*r*
Mobility	.	.	
Self-care	−4.930	<0.001 *	−0.858
Usual Activities	−4.604	<0.001 *	−0.801
Pain/Discomfort	−4.416	<0.001 *	−0.768
Anxiety/Depression	−3.755	<0.001 *	−0.653
Perceived Health Level	−5.020	<0.001 *	−0.873

* Correlation is significant at the *p* < 0.05 level.

**Table 3:** Variation analysis of the EQ-5D-5L questionnaire results—Group II.

Group II—EQ-5D-5L	Wilcoxon Test	*p*	*r*
Mobility	.	.	
Self-care	−3.938	<0.001 *	−0.656
Usual Activities	−5.097	<0.001 *	−0.849
Pain/Discomfort	−4.817	<0.001 *	−0.802
Anxiety/Depression	−3.557	<0.001 *	−0.592
Perceived Health Level	−5.094	<0.001 *	−0.849

* Correlation is significant at the *p* < 0.05 level.

**Table 4:** Correlations between variables—Group I.

			Age	Time Since Surgery	Objective Improvement (Sessions)	Total Number of Sessions	Sensory Deficit	EQ-5D-5L Perceived Health Level	Treatment Satisfaction
Spearman’s Rho	Age	Correlation Coefficient	1.000	0.217	0.150	−0.088	−0.204	−0.394 *	0.350 *
		Significance (2-tailed)	.	0.226	0.405	0.625	0.254	0.023	0.046
	Time Since Injury	Correlation Coefficient	0.217	1.000	0.300	−0.017	−0.406 *	−0.273	0.330
		Significance (2-tailed)	0.226	.	0.090	0.926	0.019	0.124	0.060
	Objective Improvement (Sessions)	Correlation Coefficient	0.150	0.300	1.000	0.203	−0.140	0.052	−0.029
		Significance (2-tailed)	0.405	0.090	.	0.258	0.436	0.772	0.875
	Total Number of Sessions	Correlation Coefficient	−0.088	−0.017	0.203	1.000	0.236	−0.034	−0.315
		Significance (2-tailed)	0.625	0.926	0.258	.	0.187	0.852	0.075
	Sensory Deficit	Correlation Coefficient	−0.204	−0.406 *	−0.140	0.236	1.000	−0.244	−0.649 **
		Significance (2-tailed)	0.254	0.019	0.436	0.187	.	0.171	0.000
	EQ-5D-5L Perceived Health Level	Correlation Coefficient	−0.394 *	−0.273	0.052	−0.034	−0.244	1.000	−0.081
		Significance (2-tailed)	0.023	0.124	0.772	0.852	0.171	.	0.654
	Treatment Satisfaction	Correlation Coefficient	0.350 *	0.330	−0.029	−0.315	−0.649 **	−0.081	1.000
		Significance (2-tailed)	0.046	0.060	0.875	0.075	0.000	0.654	.

* Correlation is significant at the *p* < 0.05 level. ** Correlation is highly significant at the *p* < 0.01 level.

**Table 5:** Correlations between variables—Group II.

			Age	Time Since Surgery	Objective Improvement (Sessions)	Total Number of Sessions	Sensory Deficit	EQ-5D-5L Perceived Health Level	Treatment Satisfaction
Spearman’s Rho	Age	Correlation Coefficient	1.000	0.281	0.269	0.197	0.195	−0.476 **	−0.374 *
		Significance (2-tailed)	.	0.097	0.113	0.250	0.254	0.003	0.025
	Time Since Injury	Correlation Coefficient	0.281	1.000	−0.190	−0.225	0.098	−0.188	−0.301
		Significance (2-tailed)	0.097	.	0.266	0.187	0.568	0.272	0.075
	Objective Improvement (Sessions)	Correlation Coefficient	0.269	−0.190	1.000	0.535 **	−0.056	0.079	0.048
		Significance (2-tailed)	0.113	0.266	.	0.001	0.744	0.645	0.779
	Total Number of Sessions	Correlation Coefficient	0.197	−0.225	0.535 **	1.000	0.248	−0.218	−0.293
		Significance (2-tailed)	0.250	0.187	0.001	.	0.145	0.201	0.083
	Sensory Deficit	Correlation Coefficient	0.195	0.098	−0.056	0.248	1.000	−0.541 **	−0.519 **
		Significance (2-tailed)	0.254	0.568	0.744	0.145	.	0.001	0.001
	EQ-5D-5L Perceived Health Level	Correlation Coefficient	−0.476 **	−0.188	0.079	−0.218	−0.541 **	1.000	0.722 **
		Significance (2-tailed)	0.003	0.272	0.645	0.201	0.001	.	0.000
	Treatment Satisfaction	Correlation Coefficient	−0.374 *	−0.301	0.048	−0.293	−0.519 **	0.722 **	1.000
		Significance (2-tailed)	0.025	0.075	0.779	0.083	0.001	0.000	.

* Correlation is significant at the *p* < 0.05 level. ** Correlation is highly significant at the *p* < 0.01 level.

## Data Availability

The datasets presented in this article are not readily available because they are part of an ongoing study and are protected by national privacy laws.
